# Coarse-Grained Prediction of RNA Loop Structures

**DOI:** 10.1371/journal.pone.0048460

**Published:** 2012-11-08

**Authors:** Liang Liu, Shi-Jie Chen

**Affiliations:** Department of Physics and Department of Biochemistry, University of Missouri, Columbia, Missouri, United States of America; University of Georgia, United States of America

## Abstract

One of the key issues in the theoretical prediction of RNA folding is the prediction of loop structure from the sequence. RNA loop free energies are dependent on the loop sequence content. However, most current models account only for the loop length-dependence. The previously developed “Vfold” model (a coarse-grained RNA folding model) provides an effective method to generate the complete ensemble of coarse-grained RNA loop and junction conformations. However, due to the lack of sequence-dependent scoring parameters, the method is unable to identify the native and near-native structures from the sequence. In this study, using a previously developed iterative method for extracting the knowledge-based potential parameters from the known structures, we derive a set of dinucleotide-based statistical potentials for RNA loops and junctions. A unique advantage of the approach is its ability to go beyond the the (known) native structures by accounting for the full free energy landscape, including all the nonnative folds. The benchmark tests indicate that for given loop/junction sequences, the statistical potentials enable successful predictions for the coarse-grained 3D structures from the complete conformational ensemble generated by the Vfold model. The predicted coarse-grained structures can provide useful initial folds for further detailed structural refinement.

## Introduction

The ability to predict RNA 3D structure is critical for understanding RNA functions. Recent developments in *de novo* prediction of RNA 3D structures have led to highly promising results [1]–[18] (for review, see [19]–[24]). In particular, several *de novo* structure prediction methods have been developed based on the knowledge-based energy function. For example, Dima and co-workers [25] extracted the base-pair stacking parameters from RNA native structures and the extracted parameters agree with the experimental data [26]. Wu et al. [27] explored the correlation between RNA secondary structural motifs and their thermodynamic stability to derive energy parameters for base-pair stackings, and free segments such as hairpin loops, internal loops and bulge loops. Bernauer and co-workers [15] further extracted a set of distance-dependent energy parameters between any two bases, irrespective of the locations of the bases (in a helix or a loop). Das and Baker [5], [9] obtained energy function – made available in the Rosetta software package – based on the base orientations and interactions. Parisien and Major [7] predicted 3D structures with a pipeline of two computer programs: MC-Fold and MC-Sym, developed based on the nucleotide cyclic motifs (NCMs). All of these methods have provided valuable insights into the correlation between loop sequence and their stability. Especially, these methods are particularly useful for selecting the most probable conformation from an ensemble of near-native structures.

A key issue in the predictions of RNA stability is how to compute the loop free energy. For hairpins and RNA secondary structures in general, the nearest neighbor model, which assumes that the total free energy is an additive sum of the free energy of each elements (base-pair stacking, loop), and other models [6], [26], [28]–[32] has enabled successful predictions for RNA structures and stabilities. In most of the existing models, loop stability is often assumed to depend on loop size, the identity of the closing base pair, the interaction of the first mismatch with the closing base pair, and an additional stabilization term for loops with GA or UU first mismatches [33]–[35]. Further detailed sequence-dependence of the loop stability has been ignored. Experimental results suggest that loop stability may be sensitive to the sequence context inside the loop. For example, for unusually stable RNA hairpin loops, Dale et al. [36] performed optical melting studies for a series of hairpins. The study led to a set of different stability parameters for different loop sequences, such as GNRA and UUCG (where N is any nucleotides and R is a purine) tetraloops, hexaloops with UU first mismatches, and hairpin loop of iron responsive element, GAGUGC, all of which are significantly more stable than other hairpin loops of the same length.

The prediction of sequence-dependent loop free energy requires a model that goes beyond simple fitting with the experimentally measured empirical parameters. The formation of the intraloop base pairs and stacks would cause significant restriction of the loop conformational space and the loop entropy. It is practically impossible to exhaustively measure the loop free energy for all the different possible intraloop contacts for the different sequences and loop lengths. Therefore, evaluation of loop free energy with an ensemble of possible intraloop base pairing/stacking interactions cannot be achieved by experiment alone. We also need a computational model. The main purpose of the present study is to develop a statistical potential model that enables predictions of loop and junction three-dimensional structures from the sequence.

In general, there are two classes of physics-based models for RNA structure prediction: molecular dynamics (For review, see [37]) and Monte Carlo simulation methods and polymer statistical mechanics methods. Molecular dynamics simulations have provided much insights into the atomic details of intraloop interactions and their contributions to the loop stabilities [1], [38]–[40]. The polymer statistical mechanical models often employ low-resolution (coarse-grained) conformational models in order to capture the complete conformational ensemble. Along this line there have been different ways to construct the low-resolution RNA structures. For example, with a knowledge-based potential, Jonikas et al. [8] developed a structure filter model (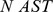) where nucleotides are represented by the 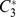 atoms. In another coarse-grained model where nucleotide are represented by the 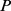 and 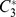 atoms, Keating and Pyle [41] developed a semi-automated approach to build RNA structures with a directed rotameric search strategy. Furthermore, in an attempt to develop a high-resolution RNA model (HiRE-RNA), Pasquali and Derreumaux [11] used six to seven beads for each nucleotide (one bead for the phosphate 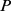, four beads for the sugar 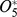, 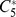, 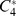, 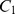, respectively, and one bead for a pyrimidine base and two beads for a purine base).

Our RNA folding model is based on a virtual bond-based RNA conformational model (called “Vfold” model; Fig. 5) [42]. The Vfold model uses two virtual bonds (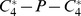) for each nucleotide and samples RNA conformations through self-avoiding walks in a diamond lattice. It provides an effective tool to sample RNA conformations and to evaluate the conformational entropy. The model has shown a high promise in predicting the 2D and 3D structures and the folding stabilities from the sequence [14], [42]–[48]. However, the Vfold model does not account for the sequence-dependent conformational propensity of the loop, namely, the model assumes that for any given loop, all the loop conformations generated in the model have the same energy. Such a simplification could cause inaccuracy in the prediction of loop stability and structure [36], [49], [50]. Physically, the sequence-dependence of loop stability arises from the local interactions, which affect the loop flexibility and hence conformational propensity [50], as well as the nonlocal interactions between the different nucleotides. In this study, we develop a method to extract a set of virtual bond-based (coarse grained) statistical potentials (scoring functions) from a set of non-redundant RNA structures such as the RNA09 database [51] and the Leontis database (http://rna.bgsu.edu/nrlist/). Specifically, we aim to derive a set of dinucleotide statistical potentials 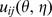 as a function of the (4
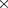
4) types of the dinucleotides base 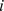 and 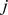 and the backbone pseudo-torsion angles (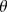, 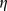) of the dinucleotide conformation. We use the RNA09 database [51], the Leontis database, the Capriotti's database [52] and the PDB database [53], [54], respectively, to test the extracted potential functions. We note that the dinucleotide in this work refers to the two continuous nucleotides within the same loop. The goal is for a given RNA loop/junction sequence, to identify the lowest-RMSD structures from the Vfold-generated complete conformational ensemble.

## Results

### Pseudo-torsion angles (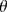, 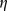)

The 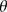 and 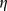 values of dinucleotides in the 152 RNA loops/junctions are plotted as a 2D scatter plot (Fig. 6), where each point represents the 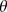-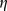 coordinates for a dinucleotide. In contrast to the analysis in the previous studies [55], [56], here we find a distinct category of dinucleotides conformation around (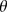 = 150, 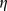 = 225). This region was once considered as the helical region, because the dinucleotides with 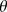-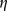 coordinates located in this group are most likely found within the RNA helix. However, as we only count the dinucleotides in RNA loops and junctions, the plot shows that the loop/junction residues can also have the tendency to have the helix-like conformation. This observation provides a rational in the next step for building the 3D all-atom structures by adding the helical residues back to the coarse-grained backbone model.

### Quasi-chemical approximation-based potentials

The 152 RNA loops/junctions within our training dataset consist of a total number of 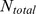 = 572 nucleotides, with the numbers of each type of the nucleotide 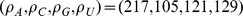 and the corresponding mole fraction of each nucleotide 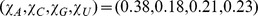.

From the dataset of the coarse-grained “correct” structures (Table 1), for each pair of the nucleotides 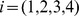 and 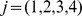 and pseudo-torsion angles (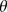, 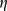), we calculate the numbers 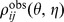 of the dinucleotides conformations and the total observed number 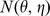 = 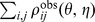. According to Eq. 8, we then calculate the expected number 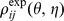 of each type of dinucleotides 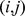 with pseudo-torsion angles (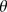, 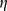). If no pseudo-torsion angles (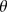,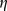) is observed for dinucleotide 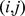, we assign an unfavorable potential 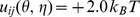 kcal/mol. From Eq. 7, we compute the potentials as a 4
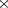
4
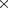
3
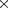
3 tensor.

**Table 1:** The number of dinucleotides with torsion angles (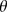, 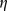).

		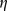		
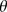		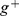	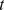	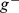
	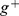	43	27	21
	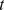	46	99	21
	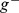	19	101	43

### Iterative approach-derived potentials

The convergence speed of the iteration depends on the selection of convergence criteria. For instance, if the convergence threshold parameter in Eq. 13 is set to 10^−3^, the iterative process would converge after around 3000 iterations. In contrast, if the convergence threshold parameter is set to 10^−2^, the iterative process would converge after 340 iterations. On an Intel(R) Xeon(R) CPU 5150 @ 2.66 GHz on Dell EM64T cluster system, the 3000-cycle iteration process took about 30 minutes and a 300-step iteration took less than 6 minutes.

Comparison between the two sets of the derived potential parameters shows that the “true” potentials 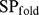 are less uniform than the “extracted” potentials (Fig. 2), suggesting that the “true” potentials 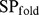 are more sensitive to the sequences and conformations of the dinucleotide and thus have the ability to discriminate the “correct” conformation from an ensemble of conformations for a given RNA loop/junction sequence.

### Tests on training datasets

We test the statistical potentials for the accuracy in loop/junction structure prediction for a large number of sequences. For a given sequence, we generate the full ensemble of the virtual bond loop/junction conformations using our Vfold model. Each conformation is then scored by the total statistical potential, which is evaluated as the sum of the statistical potentials (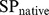 or 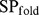) for the dinucleotide pairs in the conformation. The conformation with the lowest value of the total statistical potential is identified as the predicted native structure. If the predicted native structure is the same as the coarse-grained “correct” structure (i.e., the virtual bond structure that is closest to the PDB structure, evaluated by RMSD), the prediction is successful for the sequence; otherwise, the prediction fails.

We first apply the two sets of statistical potentials to the RNA loops and junctions in the training dataset. Such a test for structure prediction is nontrivial because the SP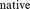 and SP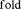 are derived based on the frequency 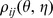 (see Eqs. 7 and 10) instead of the structure. As described above, 152 loops and junctions are constructed from the 262 RNA structures in the RNA09 dataset, including 72 RNA hairpin loops, 25 internal/bulge loops, 14 pseudoknot loops, 35 multibranched loops and 6 junctions (the free segments other than the above 4 types) (Table 3A). Here we note that the two loops in an internal loops are counted separately in our calculation, as our model does not specify specific types of loops or junctions. This rule is also applied to the pseudoknot loops and multibranched loops.

We found that 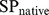 succeeded in finding 130 coarse-grained “correct” (lowest potential) structures out of 152 loops and junctions. In contrast, 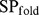 can give successful predictions for all the 152 the coarse-grained “correct” conformations (Table 3A). 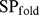 is more reliable than 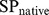 in structure prediction with success rate 100% vs 85.5%.

### Test on the Capriotti's dataset

To rigorously test the reliability of the two sets of potential parameters, we need to perform the test on loops and junctions outside the training dataset. We will perform tests for several such test sets. We first choose a dataset collected by Capriotti, et al. [52], consisting of 85 structures with length 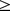
20 nucleotides and solved at resolution better than 3.5 Å. The 3DNA software determined a total of 72 loops/junctions with lengths ranging from 3 nt to 8 nt, excluding the 5′/3′-terminal dangling regions. The 72 loops/junctions can be classified into 37 hairpin loops, 14 internal/bulge loops, 5 pseudoknot loops, 1 junction and 15 multibranched loops (Table 3B).

Our results indicate that 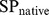 and 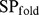 potentials can successfully find out 62 and 70 coarse-grained “correct” conformations, respectively, out of the 72 test cases (Table 3B). The result indicates that the “true” potential parameters 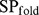 are more reliable than the directly extracted potentials 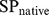 (success rate 97% vs 86%). One of the two failed predictions for 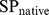 is for a pseudoknot loop, which has special loop structure due to the tertiary interactions (base triplets) with the helices. Another failed prediction is a junction located in a large RNA molecule and the junction structure is determined by not only its sequence content but also the surrounding structural environment.

### Test on the PDB dataset

The January 2012 version of PDB database contains 2227 structures that contain at least one strand of RNA sequence. These structures range from hairpin-loop structures to RNA-protein complexes or RNA-DNA hybrids. We found 8452 loops and junctions in the 2227 PDB structures (*TEST-I*). All the loops and junctions (excluding the 3′/5′-terminal dangling regions) have lengths from 3 nt to 8 nt. Within these 2227 RNA molecules, 1609 have structures determined by X-ray crystallography, which contain 7459 RNA loops and junctions (*TEST-II*), and 934 of these 1609 RNAs have high-resolution structures (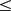
3.0 Å), which contain 1119 RNA loops and junctions (*TEST-III*).

Our test results show that the numbers of the correct predictions with the statistical potentials 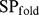 and 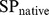 are 7364 (success rate = 87%) v.s. 6992 (success rate = 83%) for *TEST-I*, 6553 (success rate = 88%) v.s. 6222 (success rate = 83%) for *TEST-II*, and 1070 (success rate = 95%) v.s. 1001 (success rate = 89%) for *TEST-III*, respectively. Fig. 10 shows illustrations for two of the results (a hairpin loop from PDB structure 1IVS and an internal loop from PDB structure 1JJ2).

If we include the top five lowest-potential conformations, the numbers of successfully determined loops and junctions are increased to 8006 with 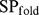 v.s. 7857 with 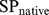 for *TEST-I*, 7089 v.s. 6949 for *TEST-II* and 1102 v.s. 1065 for *TEST-III* (Table 2). Fig. 11 shows the minimal-RMSD structure, the 9-th potential structure computed with 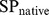, and the predicted structure with the lowest-potential, for multibranched loop from PDB structure 3CCM.

**Table 2:** The numbers of sequences with successfully predicted loop/junction structures for (I) all the 8452 RNA loops/junctions in *TEST-I* (II) the 7459 RNA loops and junctions in *TEST-II* and (III) the 1119 RNA loops and junctions in *TEST-III*.

	I		II		III	
	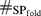	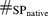	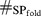	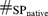	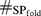	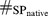
Top-1	7364	6992	6555	6222	1070	1001
Top-2	7686	7411	6819	6563	1083	1022
Top-3	7796	7715	6909	6826	1101	1060
Top-4	7956	7798	7043	6895	1102	1061
Top-5	8006	7857	7089	6949	1102	1065
TOTAL- 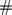	8452		7459		1119	

For each dataset, the numbers in columns are calculated with the 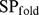 (“
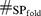
”, “true” potential parameters) and 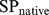 (“
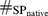
”, “extracted” potential parameters), respectively. The potentials are obtained from the RNA09 database. The “Top-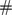
” in the first column means that the “correct” structure is in the top-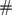 lowest-potential conformations.

Moreover, we test the predictions of the first 20 lowest-potential conformations for the loops and junctions in all the three databases *TEST-I*, *TEST-II* and *TEST-III* as well as the influence of the convergence threshold parameter on the accuracy of the structure prediction. Fig. 12 shows the numbers of successfully determined loops and junctions in the first 20 lowest-potential conformations for the three databases. The comparisons between the predicted results with 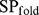 and 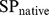 supports our conclusions in the previously two benchmark tests that 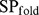 is more reliable than 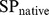. The loops/junctions that we failed to predict mostly involve tertiary interactions with helices or other cofactors such as protein and DNA.

The predicted results using the statistical potentials 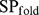 and 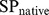, extracted from the Leontis dataset also support the above conclusions (Fig. 9 and Table 4).

Fig. 13 shows the sensitivity of the convergence threshold parameter (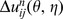 = 0.001 v.s. 0.01) to the RNA structural predictions. The comparisons of the numbers of the successfully determined loops and junctions in the three databases show that the convergence threshold parameters 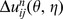 do not have strong influence on the accuracy of the structure predictions.

Furthermore, we categorize the sequences in the dataset *TEST-III* and the predicted results according to the types of RNA loops and junctions. The 1119 RNA loops and junctions can be grouped into 408 hairpin loops, 166 internal/bulge loops, 148 pseudoknot loops, 329 multibranched loops and 68 junctions. The correct predictions with 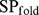 for each type of RNA loops and junctions are 395, 165, 147, 297 and 66, respectively (Table 3C). For junctions, multibranched loops, pseudoknots, internal/bulge loops and five of the hairpin loops, the failed predictions are due to the interactions beyond the dinucleotide context, such as the loop-helix tertiary interactions and RNA-protein interactions. The possible reason for other six failed predictions for hairpin loops is that these hairpin loops are not closed by canonical base pairs. These loops are closed by non-canonical pairs, such as AA or AC, which may lead to different hairpin loop structures.

**Table 3:** The types and numbers of loops and junctions in (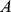) the RNA09 dataset, (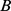) the Capriotti's dataset and (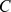) the *TEST-III* dataset, and the number of the correct predictions with the “true” potentials 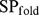.

	RNA09		Capriotti's		*TEST-III*	
Types	Total #	Correct #	Total #	Correct #	Total #	Correct #
Hairpin	72	72	37	37	408	395
Internal/bulge	25	25	14	14	166	165
Pseudoknot	14	14	5	4	148	147
Multibranched	35	35	15	15	329	297
Junction	6	6	1	0	68	66
TOTAL	152	152	72	70	1119	1070

For the *TEST-I* (the 8452 RNA structures in PDB) and *TEST-II* (the 7459 x-ray structures in *TEST-I*) databases, we randomly selected 3229 RNA loops/junctions from the database *TEST-I* and categorize them according to the types of RNA loops and junctions. The 3229 RNA loops and junctions contain 1406 hairpin loops, 255 internal/bulge loops, 184 pseudoknot loops, 1265 multibranched loops and 119 junctions. The correct (top-1) predictions with 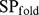 for each type of RNA loops and junctions are 1274 (success rate = 90.6%), 245 (success rate = 96.1%), 178 (success rate = 96.7%), 1162 (success rate = 91.9%) and 110 (success rate = 92.4%), respectively. The total number of the successful predictions is 2969 (success rate = 91.9%).

### Statistical potentials and the Leontis dataset

The March 17, 2012 version of the Leontis dataset contains 642 RNA sequences with structures of resolution better than 4.0 Å. The 3DNA software identified a total of 435 RNA loops and junctions with lengths ranging from 3 nt to 8 nt, excluding the 5′/3′-terminal dangling regions. Our calculations show that the difference between the diamond lattice-represented structures and the PDB structures varies for the different loop lengths and sequence contents with RMSD from 0.74 Å to 3.93 Å (Fig. 8), and the mean and standard deviation of RMSD values are 1.37 Å and 0.29 Å, respectively. We use such coarse-grained structures to calculate the observed dinucleotide frequencies, to extract 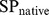 and to search for the “true” potential functions 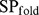.

Figs. 3 and 4 show the comparison between the potentials 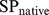/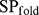 extracted from the dataset RNA09 and from the Leontis dataset. We apply the “true” potential 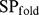 extracted from the Leontis dataset to predict the loop/junction structures in three testing datasets: *TEST-I*, *TEST-II* and *TEST-III*, constructed from the January 2012 version of PDB database. Our test results (Table 4) show a success rates of 87% (7369 out of 8452) for *TEST-I*, 88% (6567 out of 7459) for *TEST-II*, and 96% (1077 out of 1119) for *TEST-III*, respectively.

**Table 4:** The numbers of sequences of the successfully predicted loop/junction structures for (I) all 8452 RNA loops/junctions in *TEST-I* (II) the 7459 RNA loops and junctions in *TEST-II* and (III) the 1119 RNA loops and junctions in *TEST-III*.

*TEST-I*				
	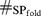 (A)	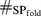 (B)	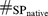 (A)	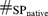 (B)
Top-1	7364	7369	6992	7102
Top-2	7686	7686	7411	7500
Top-3	7796	7862	7715	7665
Top-4	7956	7969	7798	7844
Top-5	8006	8007	7857	7878
TOTAL- 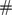	8452			

For each dataset, the numbers in columns “A” are calculated from 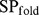 (“
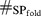
”, “true” potential parameters) and 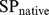 (“
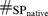
”, “extracted” potential parameters), obtained from the RNA09 database, and the numbers in columns “B” are calculated from 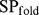 (“
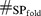
”) and 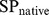 (“
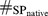
”), obtained from the Leontis' database respectively. The “Top-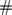
” in the first column means that the “correct” structure is in the top 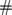 lowest-potential conformations.

Moreover, the predictions of the top 20 lowest-potential conformations for the loops and junctions in all the three test datasets, *TEST-I*, *TEST-II* and *TEST-III*, are shown in Fig. 9, with comparisons with the predictions based on the 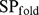 from the RNA09 dataset. The comparisons for the three test datasets (Table 4 and Fig. 9) show that the two “true” statistical potentials 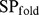 extracted from the RNA09 dataset and the Leontis dataset lead to similar success rates in structure prediction and the predictions are not sensitive to the choice of the specific training set.

## Discussion

Motivated by the biological significance to predict sequence-dependent loop and junction structures, we have developed a knowledge-based scoring functions/potentials to predict the structures of RNA loops and junctions. We use a coarse-grained conformational model (the virtual bond model) to sample RNA loop/junction conformations. From the known RNA structures, we extract a set of sequence-dependent dinucleotide-based statistical potentials using two methods. In the first method, the statistical potentials (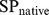) are derived from the distributions of the dinucleotide conformations in the known (native) structures. In the second method, the statistical potentials (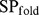) are derived based on the folding stability of the native structures (against all the other nonnative folds).

For a given sequence, the extracted statistical potentials enable ranking of the different conformations with the top ranked (the lowest-potential) structure as the predicted native structure. Extensive tests indicate that the statistical potentials can successfully predict the native structure for a large class of loop and junction sequences and that 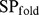 consistently outperforms 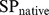 in structure prediction. Our test results also indicate that our results are not sensitive to the choice of the specific training dataset (Fig. 9 and Table 4).

The present approach has several advantages. First, the extraction of the statistical potential 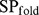 is based on the sampling of the complete conformational ensemble, including the native and all the nonnative folds. Second, because we consider all the nonnative folds in the derivation of the statistical potential, our statistical potentials can be used to predict folding from the sequence. The build-up strategy for RNA loop structure in this study is a de novo approach. Compared with other 3D loop structure prediction models, such as ModeRNA [22] and RLooM [50], our loop/junction structure prediction method is based on the complete ensemble of the (coarse-grained) conformations and does not rely on the information of template structures or homologous RNA structures. The only input information for the prediction is the sequence. Therefore, the model can predict the low-resolution structure from the sequence if no known homologous conformations can be found in the PDB. With the predicted low-resolution scaffold, one may further predict the all-atom structures using the all-atom potential methods that are derived based on near-native structures [5], [15]. The present coarse-grained model offers a useful complement to the other all-atom based models.

We have performed extensive benchmark tests using the different databases. However, a direct comparison between our model other methods is not very straightforward. This is because our model is a coarse-grained model while other models mainly focus on the all-atom structures. Furthermore, our model aims to fold a low-resolution structure from the sequence without using any input information such as homologous templates, while other models mainly focus on the prediction of the native structures from near-native folds. Future development of our method, which may give all-atom structures from the low-resolution folds, would make direct comparison between our model and other models possible.

Applications of the present statistical potentials to the Vfold structure prediction model [14] may provide an effective strategy for better structure prediction. Despite the success, there are several limitations of our model. First, the current study is based on the coarse-grained Vfold model, which only provides a low-resolution approximation for the all-atom structure of RNA loops and junctions. Ultimately an all atom-based model is required to treat the detailed tertiary interactions. Future development of the model should address the issue to build all-atom RNA structures based on the low-resolution (Vfold-generated) representations. Second, the statistical potentials are derived for dinucleotide conformations. In realistic loop and junction structures, the long-range sequence effects within the loop, such as intra-loop interactions, can influence the loop structure. The present model cannot explicitly account for such long-range intraloop interactions, which occur frequently in loops and between the different loops. Following the same procedure outlined above, one may develop a trinucleotide or higher order many-body statistical potentials to address this issue. Third, in the current form of the model, we use the same set of statistical potentials for the different types of loops/junctions. More refined potentials according to RNA loop/junction types may lead to further improvement of the accuracy of model.

## Materials and Methods

### Vfold model

We use the Vfold model to generate the full conformation ensemble of a given RNA loop or junction sequence. The Vfold model is a virtual bond-based RNA folding model [42], [45], [48], which is developed based on the two observations: the 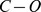 torsion in the nucleotide backbone of RNA tend to adopt the 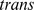 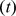 rotational isometric state (Fig. 5A) and the 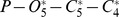 bonds and the 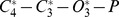 bonds in the nucleotide backbone are approximately planar [57]. Therefore, the nucleotide backbone conformations can be reduced into two effective virtual bonds 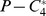 and 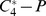 [58]–[60]. The length of each backbone virtual bond is about 3.9 Å. The virtual bonds show rotamer-like configurations gauche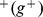, trans 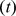 and gauche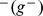 (Fig. 5C) [55], [56], [61]. Such virtual bond conformations can be well represented by the bonds in a diamond lattice. Therefore, we can use self-avoiding walks on a diamond lattice to enumerate the conformations of RNA sequences. This approach promises proper treatment of the excluded volume effect between the different atoms and the complete sampling of the conformational ensemble.

The loop structures are enumerated on a diamond lattice through exhaustive self-avoiding walks. If the first atom (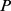) is fixed at position 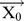, then the coordinates (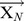) of 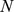-th atom (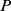 or 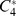) can be calculated with
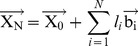
(1)where, 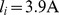 is the bond length of virtual bond 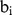 and 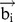 is the unit vector of virtual bond 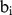. Also, the relation between the (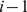)-th virtual bond (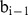) and the 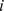-th virtual bond (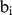) is:
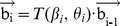
(2)where, 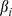 and 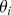 are the related bond angles 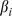 = 120 and pseudo-torsion angle 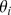 = 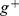 (60), 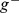 (300) or 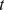 (180) (Fig. 5C). The matrix 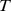 is defined as
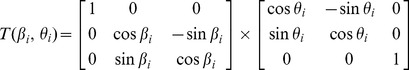
(3)Therefore, if the unit vector of the first virtual bond (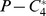) is 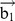, we can obtain the unit vector of the 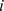-th virtual bond (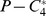 or 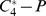) with
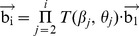
(4)


By considering excluded volume effect, i.e., two atoms cannot occupy the same site on a diamond lattice, we can generate the atomic coordinates using equations 1, 2, 3 and 4 and the conformation ensemble of RNA loops; see Ref. [42] for the detailed calculations. Here we give an example for illustration. In Fig. 5, following the 5′
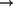
3′ direction, if the first atom 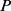 is fixed at 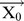 = col (0, 0, 3.9) (Å), the second atom 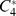 is fixed at 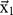 = col (0, 0, 0) (Å), then, 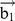 = col (0, 0, −1). According to Eq. 1, the coordinate of the third atom 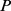 can be computed:
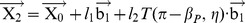
where 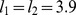 Å are the bond lengths, 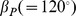 is the bond angle and 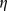 is the torsion angle (we select 180 for illustration). According to Eq. 3, the matrix is computed as:
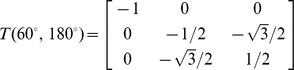
Therefore, the coordinate of the third atom 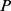 is col (0, 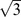/2, −1/2) (3.9 Å). Following the procedure and considering the excluded volume between any two atoms, we can compute the coordinates for other atoms and generate the conformations for a given RNA loop.

### Statistical potential

Assuming an equilibrium Boltzmann distribution for the structures in the PDB [53], [54] and NDB [62] database, one can extract the interaction potentials:
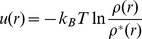
(5)Here, 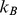 is the Boltzmann constant, 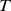 is the absolute temperature, 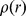 is the observed density, and 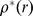 is the density in a “reference” state where no interactions occur.

The above approach can give continuous distance-dependent pairwise potentials [15], [63]–[66] or discrete base pairs/stacks potentials [27], [67]. In this work, we extract the potentials for the different dinucleotide conformations described by the pseudo-torsion angles 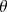 (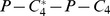) and 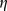 (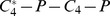) and the different dinucleotide sequences ( Fig. 5). The potential 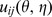 is extracted based on the following formula:
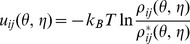
(6)where 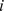 and 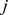 denote the types of the nucleotides (bases) of the two consecutive nucleotides within the same loop and (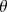, 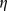) are the pseudo-torsion angles of the dinucleotide.

### Reference state

The proper choice of the reference state is a key issue in deriving the statistical potentials from the known structures. As pointed out by Thomas and Dill [68], [69], an accurate ideal reference state is not achievable. Different approximations such as the quasi-chemical approximation [64] have been used to model the reference state. Furthermore, to circumvent the reference state problem, an iterative method for successive refinement of the statistical potentials was developed and was shown to give reliable scoring functions for protein folding [68], [69] and protein-ligand interactions [63]. Here, for comparison, we employ the above two approaches to extract two different sets of RNA knowledge-based potentials and apply the extracted potentials to RNA loop and junction structure prediction.

#### Quasi-chemical approximation-based approach

In the quasi-chemical approximation [64] we use an “expected state” as the “reference state”. Such an approximation leads to the Boltzmann relation (Eq. 6):
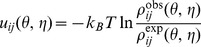
(7)where 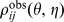 and 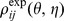 are the observed number and the expected number, respectively, of the dinucleotides (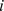, 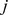) with pseudo-torsion angles (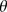, 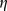) in the entire training dataset. 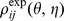 is computed from the following formulas:
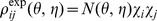
(8)where 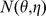 is the total observed number of dinucleotides with pseudo-torsion angles 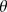 and 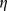 (calculated with a composition-independent scale, meaning each nucleotide is independent to other nucleotides in the state (Eq. 8)) [70], and 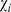 and 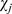 are the mole fraction of nucleotide 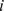 and 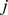 of the sequences in the entire training dataset, respectively [70].

The quasi-chemical approximation-based statistical potentials are the “extracted” energy-like parameters derived from the observed density in the database of native structures [68]. We denote such extracted statistical potentials as 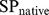. The 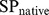 parameters are not the “true” potentials extracted from the criteria of identifying the “correct”/native structure from an ensemble of the nonnative conformations. We denote the potentials that can account for the nonnative conformations as the “true” statistical potentials (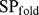).

#### Convergent iterative approach

To extract the “true” energy (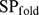), Thomas and Dill developed an iterative approach based on the folding stability of the native structure [68], [69]. The method has the advantage of accounting for the effect of the distribution of the whole conformational ensemble, including both the native and the nonnative states, for a given sequence. The overall strategy of the iterative approach is to train a set of potential parameters iteratively until the collection of the native structures in the training dataset and the full conformational ensemble (native and nonnative) lead to the same frequencies of the different dinucleotide conformations [63], [68], [69].

In our calculation, the iterative process starts with a set of initial values for the potentials, 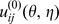. The superscript (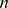) denotes the 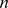-th iterative step. We use the quasi-chemical approximation-based statistical potentials 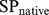 as the initial input.

At each step, we compute the total potential/score for each conformation of loop/junction by summing over the potential of all the dinucleotides contained in the loop/junction:
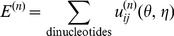
(9)Then the predicted frequencies (numbers) of the different dinucleotide conformations are computed from the Boltzmann average over all the conformations:
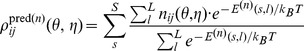
(10)where 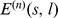 is the potential/score for the 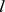-th conformation of the 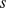-th RNA loop/junction sequence, computed with the potentials 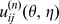 at the 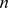-th step (Eq. 9), 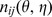 is the number of dinucleotide (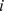, 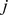) with pseudo-torsion angles (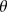, 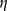) in the 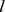-th conformation of the 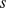-th RNA loop/junction, 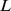 is the number of conformations of the 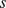-th RNA loop/junction in the training dataset, and 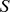 is the number of RNA loop/junction sequences in the training dataset.

In each step, we also calculate the difference between the observed and predicted dinucleotide frequencies:
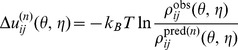
(11)where 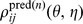 is calculated by Eq. 10 and 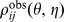 is the dinucleotide frequency observed from the “correct” (native) structures in the training dataset.

In general, the initially guessed potential parameters are not equal to the “true” potentials and thus there are differences between the observed dinucleotide frequencies and the predicted dinucleotide frequencies (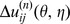 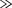
0). For each 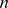-th step, we refine the potentials by accounting for the differences between the “observed” and the “predicted” frequencies of the dinucleotide conformations (see the parameter 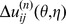 in Eq. 11):
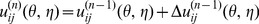
(12)We repeat the above iterative process until 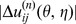 approaches 0 (smaller than a threshold number, e.g. 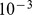). The final set of potentials 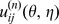 is the “true” potentials 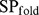.

### Flowchart

**Figure 1:**
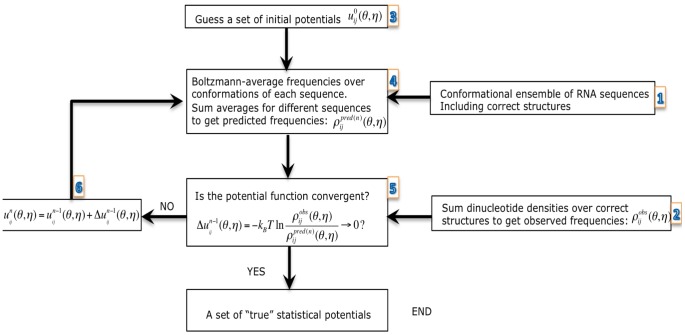
Flow chart of the iterative approach.

The iterative method is summarized as follows ( Fig. 1):

1. Prepare the training dataset of the native structures. Download RNA structures from PDB database and extract the loops and junctions. For each RNA loop/junction in the training set, generate the full conformational ensemble of RNA backbone by self-avoiding exhaustive walks on a diamond lattice (Vfold model). The conformation ensemble is used in the iterative calculation.
2. Calculate the observed dinucleotide frequencies 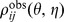 by summing the dinucleotide densities observed in the training dataset.
3. Choose a set of initially guessed potentials. In this work, we start with the “extracted” quasi-chemical approximation-based potentials (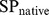).
4. Calculate the potential/score for each generated conformation of each RNA loop/junction sequence by using Eq. 9 with iterative potentials 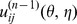. Then calculate the weighted predicted frequencies 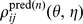 of each dinucleotide (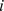, 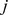) with pseudo-torsion angles (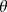, 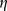), by employing Eq. 10.
5. Calculate the convergence parameter 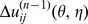 according to Eq. 11. If the following convergence condition is satisfied:
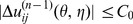
(13)with a preset small value of the threshold parameter 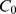, say, 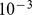, then skip to the final step and the “true” potentials 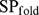 are obtained; otherwise, continue to the next step.
6. Adjust the current potentials 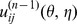 by adding the correction 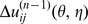 (Eq. 12), and obtain a new set of potentials 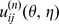. Move to the next (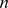-th) iterative step and return to Step 4 for the next cycle.

**Figure 2:**
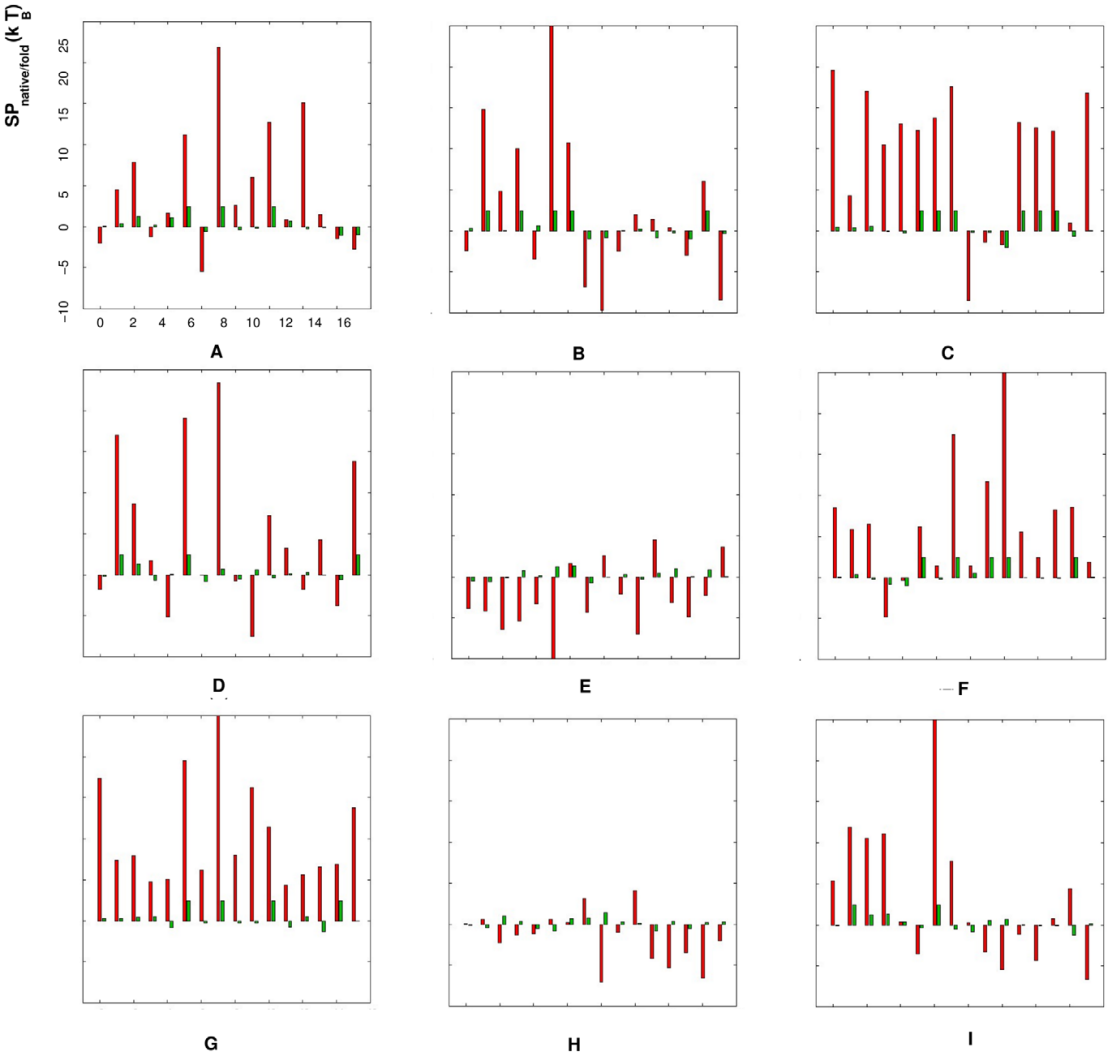
Comparison between the “extracted” statistical potentials 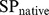 and the “true” potentials 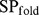. The figures from (A) to (I) stand for the torsion angles (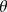, 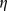 = ): (A) (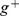, 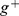), (B) (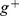, 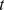), (C) (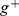, 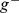), (D) (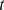, 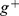), (E) (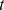, 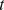), (F) (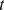, 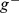), (G) (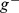, 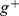), (H) (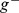, 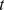) and (I) (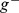, 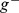), respectively. In each figure, the red bars represent the statistical potentials 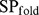, the green bars represent the statistical potentials 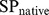, both of which are obtained from the RNA09 dataset, and the x-axis stands for the dinucleotides with different nucleotides (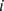, 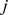 = ) AA, AC, AG, AU, CA, CC, CG, CU, GA, GC, GG, GU, UA, UC, UG and UU from 1 to 16.

**Figure 3:**
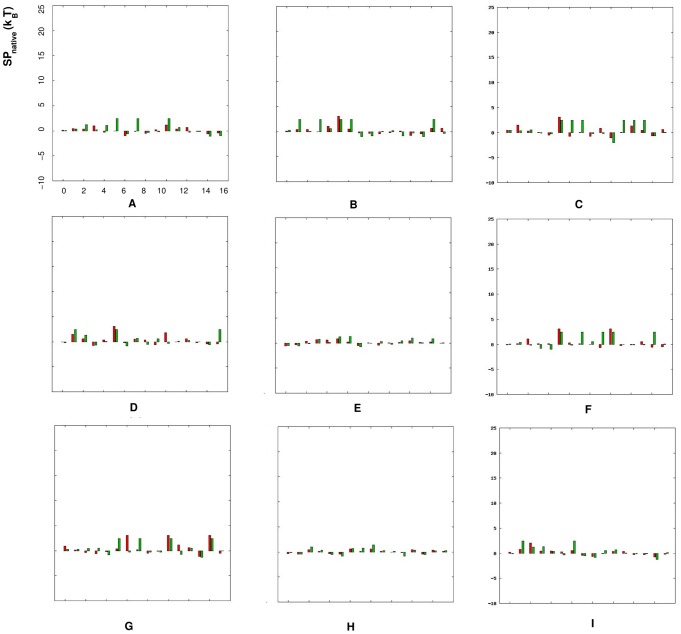
Comparison between the “extracted” statistical potentials 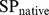 from the RNA09 dataset and the Leontis dataset, respectively. The figures from (A) to (I) stand for the torsion angles (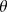, 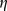 = ): (A) (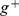, 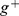), (B) (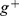, 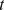), (C) (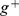, 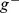), (D) (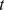, 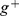), (E) (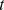, 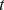), (F) (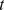, 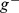), (G) (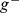, 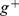), (H) (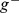, 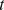) and (I) (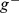, 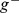), respectively. In each figure, the red bars represent the statistical potentials 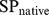 extracted from the Leontis dataset, the green bars represent the ones from the RNA09 dataset, and the x-axis stands for the dinucleotides with different nucleotides (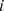, 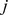 = ) AA, AC, AG, AU, CA, CC, CG, CU, GA, GC, GG, GU, UA, UC, UG and UU from 1 to 16.

**Figure 4:**
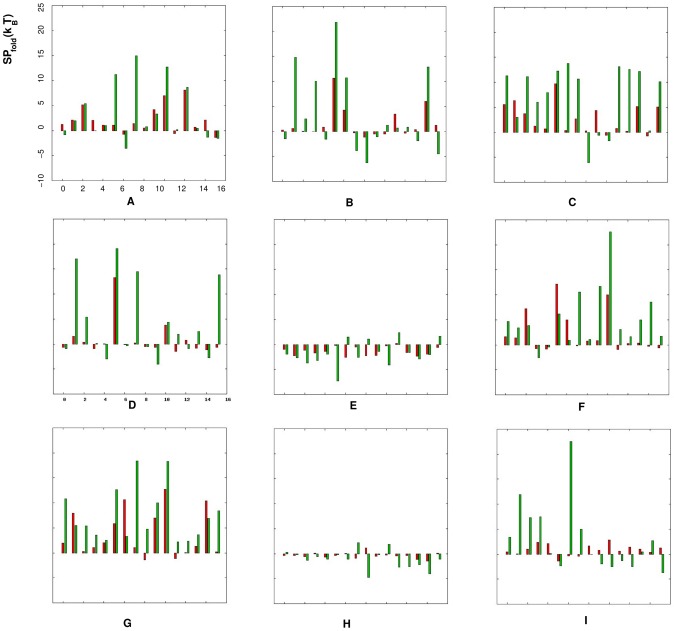
Comparison between the “true” statistical potentials 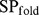 obtained from the RNA09 dataset and the Leontis dataset, respectively. The figures from (A) to (I) stand for the torsion angles (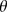, 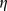 = ): (A) (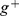, 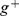), (B) (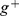, 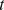), (C) (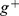, 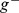), (D) (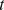, 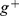), (E) (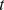, 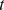), (F) (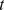, 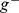), (G) (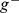, 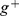), (H) (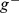, 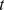) and (I) (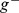, 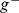), respectively. In each figure, the red bars represent the statistical potentials 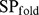 extracted from the Leontis dataset, the green bars represent the ones from the RNA09 dataset, and the x-axis stands for the dinucleotides with different nucleotides (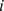, 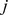 = ) AA, AC, AG, AU, CA, CC, CG, CU, GA, GC, GG, GU, UA, UC, UG and UU from 1 to 16.

**Figure 5:**
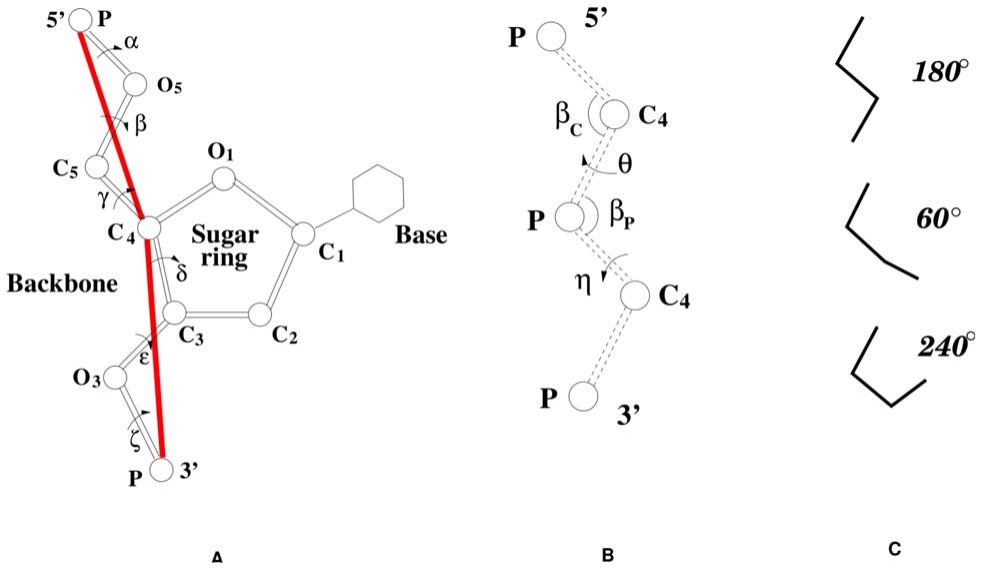
The pseudo-torsional angles. (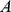) The virtual bond scheme for RNA nucleotides. (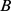) The bond angles (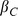, 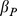) and the pseudo-torsional angles (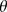, 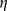) of the virtual bonds. (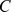) The three preferred rotamer-like configurations of the virtual bonds: 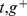 and 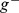, with the torsional angles equal to 180, 60 and 300, respectively.

**Figure 6:**
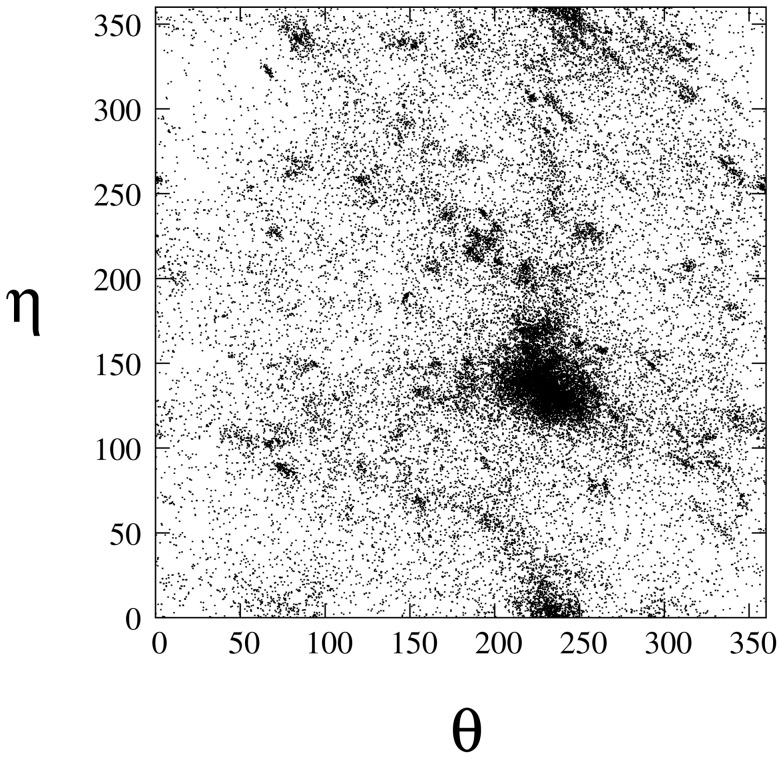
The distribution of the 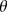 and 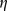 values for the different types of dinucleotides in RNA loops and junctions. The RNA loops and junctions are selected from the RNA09 dataset and the angles are calculated based on the PDB structures.

### Preparation of the training dataset

We use the RNA 2009 database (RNA09; http://Kinemage.biochem.duke.edu/databases/rnadb.phb, an updated version of previous 2005 database (RNA05) [51]) to extract the 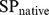 potential and use the database as the training dataset to search for the “true” potential 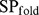. The RNA09 dataset consists of 262 RNA sequences with the experimentally determined structures of resolution 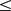
3.0 Å.

RNA loops and junctions are constructed by all the unpaired nucleotides in RNA structure. We detect the loops and junctions by removing the nucleotides involved in the base pairs. Furthermore, we remove all the loop structures with modified bases or non-RNA atoms and molecules. Advances in the knowledge of the structures of both the canonical (Watson-Crick and wobbles) base pairs and non-canonical base pairs (normally classified as tertiary interactions) [71], [72] enable the development of several automated tools to detect the base pairs in RNA structures. In this work, we use the 3DNA software package (http://3dna.rutgers.edu/home) [73] to search for all the possible base pairs from the RNA structures.

In addition, the time consumption to enumerate the loop conformations on a diamond lattice increases exponentially with the length of the loop [45]. Therefore, we only choose loops/junctions with length 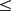
8 nt in the dataset due to the long computer time for the exhaustive enumeration of the loop conformations for longer loops [45]. In this study, we use the algorithm reported in Ref. [74] to search for the best fits on diamond lattice for the RNA loops/junctions.

Larger loops often involve tertiary interactions with other subunits of RNA structures, which are not accounted for in this study. As a result, we found 152 loops/junctions with the length ranging from 3 nt to 8 nt in the RNA09 dataset. These loop and junction structures are used as the training dataset in our iterative approach.

To further test the influence of the use of the different training dataset on the statistical potentials and the predictive power of the statistical potentials, we also use another non-redundant high-resolution RNA structure dataset, collected by Leontis' lab (http://rna.bgsu.edu/nrlist/).

### Conformational ensembles of RNA loops/junctions

We use the Vfold model to generate the full conformation ensemble of a given RNA loop or junction sequence. In the Vfold-generated loop/junction conformational ensemble, because each of the two pseudo-torsion angles (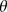 and 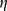) of a dinucleotide occupies three torsional states in a diamond lattice, there exist 4
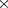
4
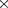
3
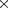
3 parameters for the potentials for the four types of each nucleotide (A, C, G and U) and the three possible rotamer-like states for each torsional angle (and hence 3
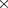
3 types of the pseudo-torsion angle pairs 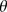 and 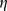).

**Figure 7:**
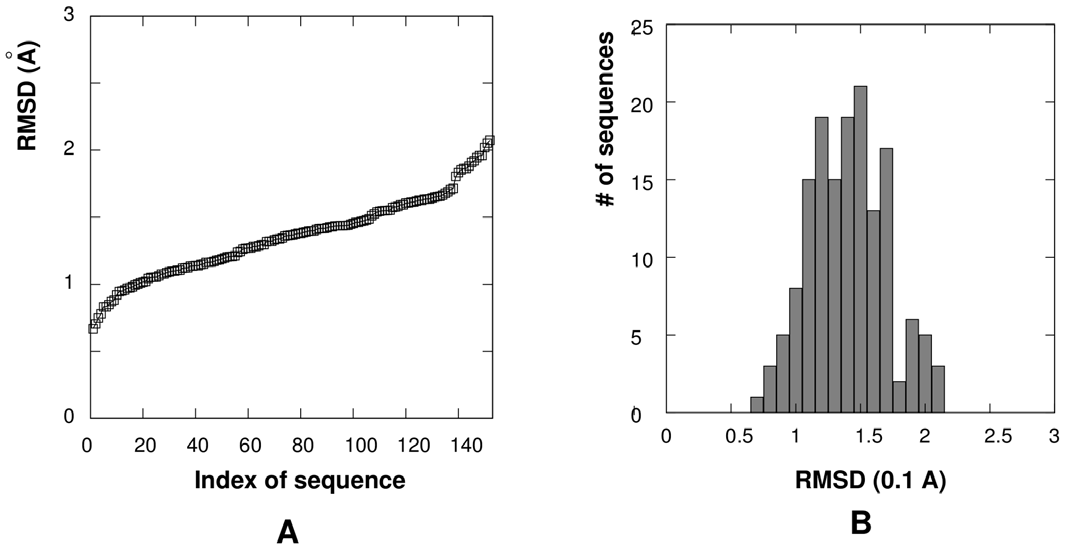
(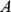) The RMSD between the PDB structures and the diamond lattice-represented structures for RNA loops and junctions in the RNA09 dataset. The x-axis represents the index of RNA loops/junctions in the RNA09 dataset. (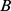) The number of RNA loops/junctions within each RMSD-value bin (0.1 Å). The mean and standard deviation of RMSD values for the 152 RNA loops/junctions are 1.35 Å and 0.30 Å, respectively.

For each PDB structure of the loop/junction, we find the minimum-RMSD fit of the virtual bond conformation on the diamond lattice. We call such a coarse-grained (correct) structure as the “coarse-grained correct structure”; see Step 1 in Fig. 1. Our calculations show that the differences between the diamond lattice-represented structures and the PDB structures varies for the different loop lengths and sequence contents with RMSD in the range from 0.67 Å to 2.07 Å (Fig. 7), the mean and standard deviation of RMSD values are 1.35 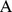 and 0.30 Å, respectively.. We will use the coarse-grained correct structure to calculate the observed dinucleotide frequencies, from which 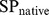 and 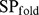 are extracted.

**Figure 8:**
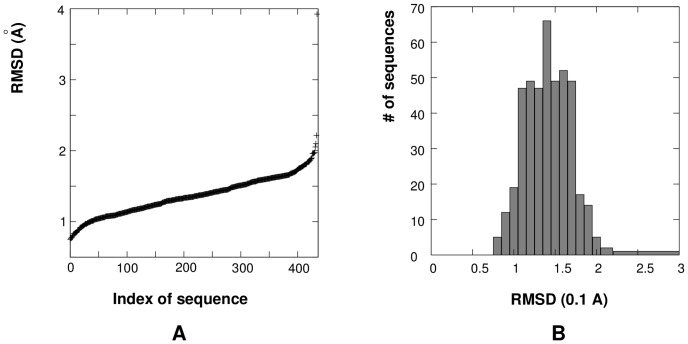
(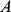) The structural differences (RMSD values) between PDB structures and diamond lattice-represented structures for the RNA loops/junctions within the Leontis dataset. The x-axis represents the index of RNA loops/junctions in the Leontis dataset. (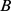) The number of RNA loops/junctions within each RMSD-value bin (0.1 Å). The mean and standard deviation of RMSD values for the 435 RNA loops/junctions are 1.37 Å and 0.29 Å, respectively.

**Figure 9:**
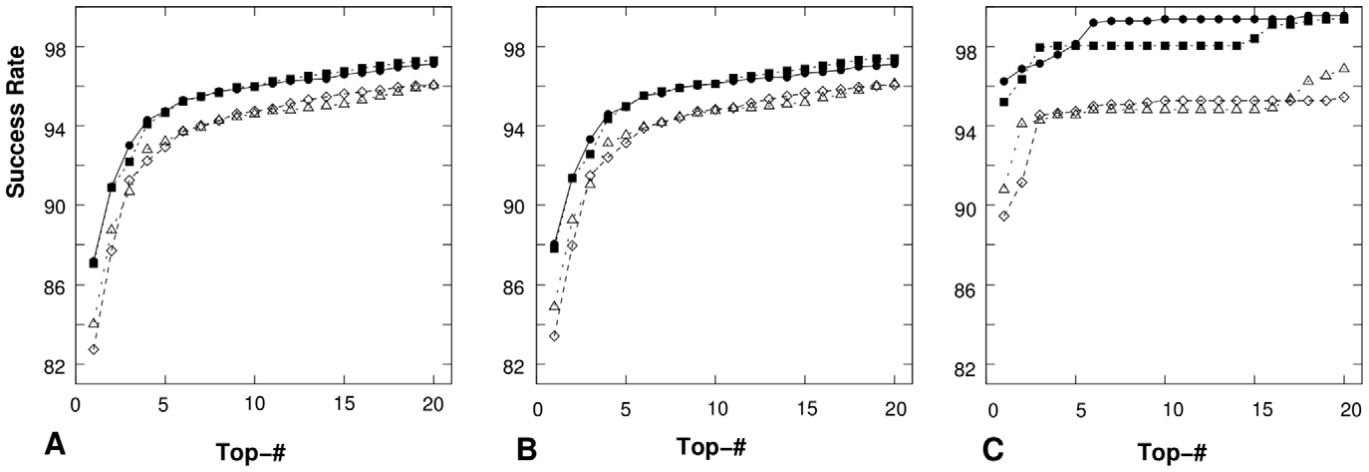
The success rate for the prediction of the coarse-grained correct loop/junction structures for (A) all the 8452 RNA loops and junctions in *TEST-I*, (B) the 7459 RNA loops and junctions in *TEST-II* and (C) the 1119 RNA loops and junctions in *TEST-III*. The “Top-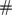
” in x-axis means that the “correct” structure is in the top 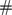 lowest-potential conformations. In each figure, “
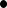
” and “▪” represent the success rate with 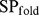 extracted from the Leontis' database and the RNA09 dataset, respectively; while, “
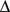
” and “◊” represent the success rate with 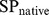 extracted from the Leontis' database and the RNA09 dataset, respectively.

**Figure 10:**
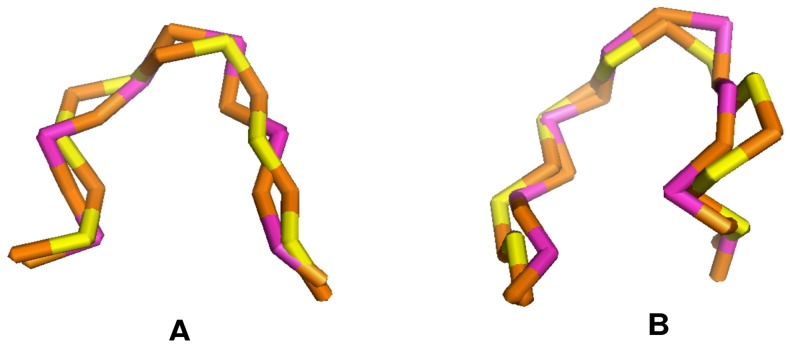
Comparison between the virtual bond-based PDB structure and correctly predicted structure with statistical potentials 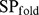
 **.** The loops are (A) a hairpin loop from PDB structure 1IVS and (B) an internal loop from PDB structure 1JJ2, respectively. In both figures, the structures shown in brown (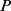) and yellow (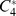) stand for the PDB structure, and the ones shown in brown (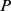) and purple (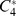) represent the correctly predicted structure, which have the lowest potential and the minimal RMSD. The RMSD values are (A) 1.52 Å and (B) 1.96 Å, respectively. The atomic structures are illustrated with Pymol software (http://www.pymol.org/).

**Figure 11:**
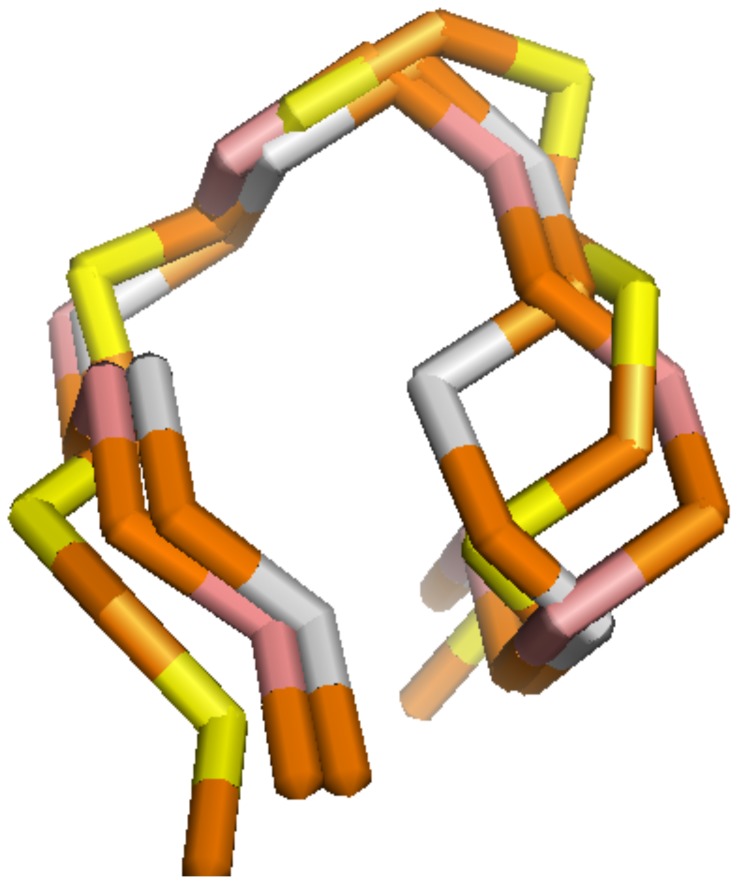
Comparison between the virtual bond-based PDB structure and top-ranked predicted structures with statistical potentials 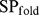
 **.** The loop is a multibranched loop from PDB structure 3CCM. In the figure, the structure shown in brown (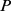) and yellow (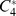) stands for the PDB structure, the one shown in brown (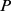) and pink (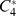) represents the minimal-RMSD structure (RMSD = 2.42 Å), and the one shown in brown (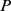) and lightgray (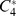) represents the predicted structure with lowest potentials (RMSD = 2.87 Å), computed with statistical potentials 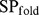. The minimal-RMSD structure has the 9-th lowest potential. The atomic structures are illustrated with Pymol software (http://www.pymol.org/).

**Figure 12:**
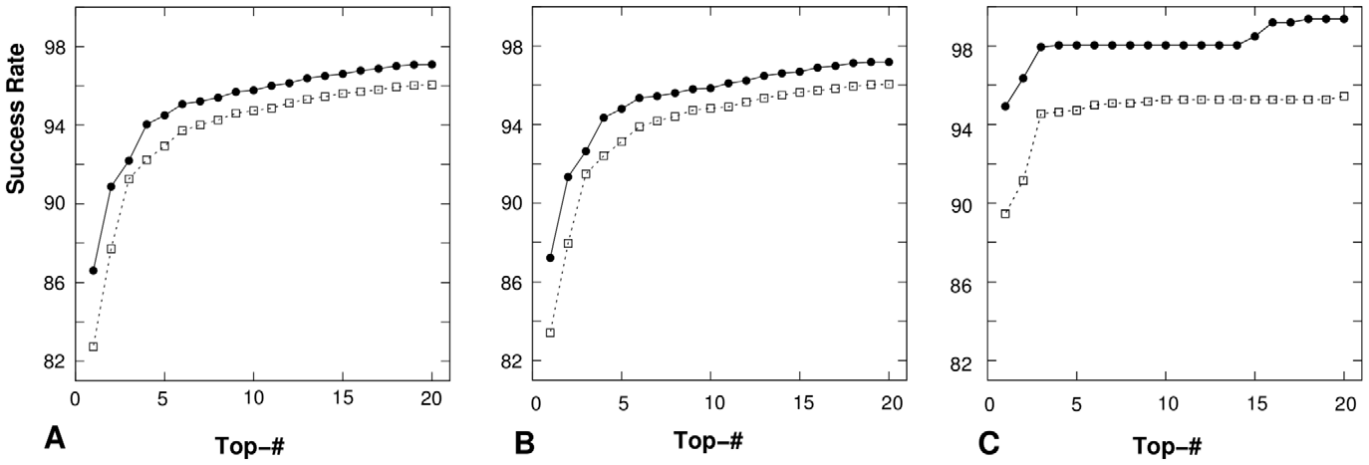
The success rate of coarse-grained correct loop/junction structure predictions for (A) all the 8452 RNA loops/junctions in *TEST-I*, (B) the 7459 RNA loops and junctions in *TEST-II*, and (C) the 1119 RNA loops and junctions in *TEST-III*. The “Top-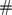
” in x-axis means that the “correct” structure is in the top 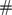 lowest-potential conformations. In each figure, 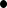 and 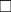 represent the success rate with 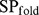 and 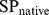, respectively.

**Figure 13:**
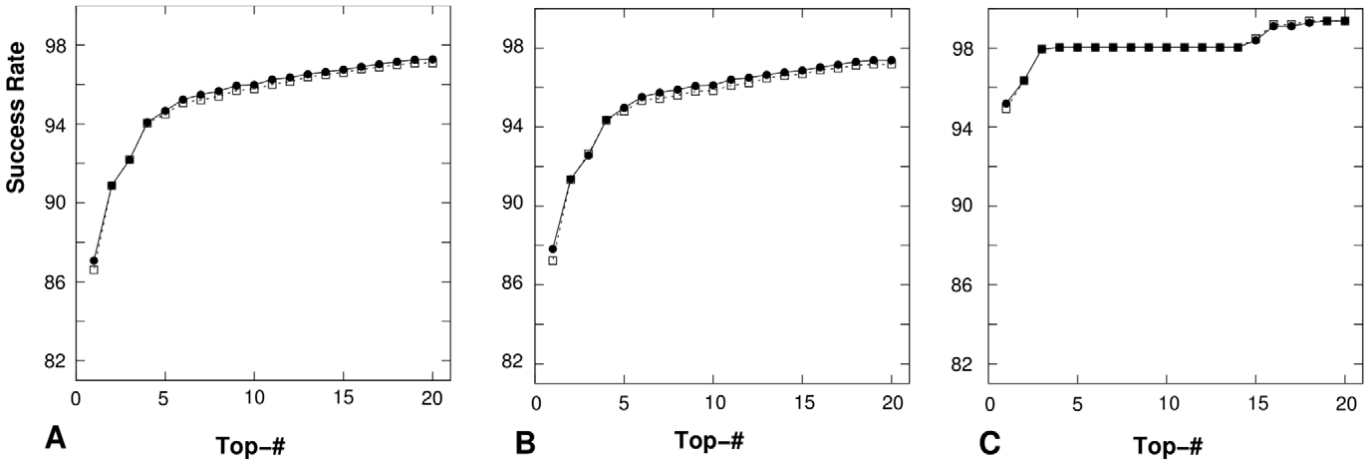
The sensitivity of the structure prediction to the change of the convergence threshold parameter : 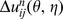 = 0.001 v.s. 0.01. The tests are based on the predictions of the RNA loops and junctions in all the three databases: (A) *TEST-I*, (B) *TEST-II* and (C) *TEST-III*. In each figure, 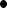 and 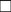 represent the success rate of the 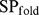-based structure prediction with 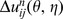 = 0.001 and 0.01, respectively.
